# Prevalence and Molecular Characteristics of Waterborne Pathogen Legionella in Industrial Cooling Tower Environments

**DOI:** 10.3390/ijerph121012605

**Published:** 2015-10-12

**Authors:** Lijie Li, Tian Qin, Yun Li, Haijian Zhou, Hongmei Song, Hongyu Ren, Liping Li, Yongguang Li, Dong Zhao

**Affiliations:** 1 Shijiazhuang Center for Disease Control and Prevention, No.3, Likang Street, Shijiazhuang 050011, China; E-Mails: eryanghualv@aliyun.com (L.L.); songhm1225@sohu.com (H.S.); jinhuangse913@sohu.com (L.L.); 2 State Key Laboratory for Infectious Disease Prevention and Control, National Institute for Communicable Disease Control and Prevention, Chinese Center for Disease Control and Prevention, No. 155, Changbai Road, Changping, Beijing 102206, China; E-Mails: qintian@icdc.cn (T.Q.); zhj_0901@163.com (H.Z.); hongyu2690@sohu.com (H.R.); 3 Collaborative Innovation Centre for Diagnosis and Treatment of Infectious Diseases, Hangzhou 310003, China; 4 Hebei International Travel Health-Care Center, No.409-1, Hongqi Stree, Shijiazhuang 050000, China; E-Mail: liyun0302@aliyun.com; 5 Shijiazhuang Jiyuan Environmental Protection Technology Co.Ltd, Shijiazhuang 050035, China; E-Mail: 13933191875@126.com

**Keywords:** aquatic bacteria, *Legionella*, *Legionella* monitoring, cooling tower water, molecular characteristics, water pollution

## Abstract

Cooling towers are a source of Legionnaires’ disease. It is important from a public health perspective to survey industrial cooling towers for the presence of *Legionella*. Prospective surveillance of the extent of *Legionella* pollution was conducted at factories in Shijiazhuang, China between March 2011 and September 2012. Overall, 35.7% of 255 industrial cooling tower water samples showed *Legionella-*positive, and their concentrations ranged from 100 Colony-Forming Units (CFU)/liter to 88,000 CFU/liter, with an average concentration of 9100 CFU/liter. A total of 121 isolates were obtained. All isolates were *L. pneumophila*, and the isolated serogroups included serogroups 1 (68 isolates, 56.2%), 6 (25, 20.7%), 5 (12, 9.9%), 8 (8, 6.6%), 3 (6, 5.0%) and 9 (2, 1.6%). All 121 isolates were analyzed by pulsed-field gel electrophoresis (PFGE) and 64 different patterns were obtained. All 121 isolates were analyzed sequence-based typing (SBT), a full 7-allele profile was obtained from 117 isolates. One hundred and seventeen isolates were divided into 49 sequence types. Two virulence genes, *lvh* and *rtxA*, are analyzed by polymerase chain reaction (PCR). 92.6% (112/121) and 98.3% (119/121) isolates carried *lvh* and *rtxA* respectively and 90.9% (110/121) of tested isolates carried both genes. Our results demonstrated high prevalence and genetic polymorphism of *L. pneumophila* in industrial cooling tower environments in Shijiazhang, China, and the SBT and virulence gene PCR results suggested that the isolates were pathogenic. Improved control and prevention strategies are urgently needed.

## 1. Introduction

*Legionella*, the aetiologic agents of legionellosis, are ubiquitous worldwide in rivers and lakes [1] and man-made water systems such as spas and cooling towers [2]. To date, more than 50 *Legionella* species have been characterized, and 25 species are known to cause human disease [2,3]. Most human infections are caused by *Legionella pneumophila*, which is responsible for approximately 90% of the identified clinical cases, and the predominant serogroup is serogroup 1 [2,4,5,6].

Transmission of bacteria from the environment to humans occurs via inhalation or aspiration of *Legionella*-containing aerosols [7,8]. Cooling towers that are contaminated by *Legionella* have been identified as the cause of sporadic cases and outbreaks of legionellosis among people living nearby [9,10]. It is important from a public health perspective to survey industrial cooling towers systems for the presence of *Legionella* [11,12].

Several outbreaks and a number of cases of *Legionella* infection that were associated with industrial cooling towers have been reported worldwide [13,14,15]. In China, industrial cooling towers are widely distributed. In our previous study that compared the ability of four methods, culture, polymerase chain reaction (PCR), quantitative real-time PCR (qPCR) and Ethidium monoazide (EMA)-quantitative real-time PCR (EMA-qPCR), to detect *Legionella* in different types of water systems, we isolated *Legionella* from cooling towers at a rate of up to 31.0% by quantitative real-time PCR. When using the culture method, the percent-positive rate for *Legionella* was 26.4%, and the concentrations of *Legionella* were as high as 1500 Colony-Forming Units (CFU)/liter [16]. These results showed high degree of pollution of *Legionella* in cooling towers water samples in China. However, there is no surveillance system focusing on the *Legionella* in the industrial cooling towers.

The industrial cooling towers are usually located in the factories; the arrangement of industrial cooling towers is different to that in other public places. In other public places, such as buildings, hospitals and hotels, the cooling towers are located on the roof, where few people access. But in the factories, the cooling towers are located on the edge of the road, where people often go through. Furthermore, the circulating water in cooling towers can be aerosolized. All these factors increase the risk of *Legionella* infection. It is important to survey the contamination of *Legionella* in cooling towers to avoid serious outbreaks of Legionnaires’ disease.

To systematically investigate the presence, population structure and infection potential of *Legionella* in industrial cooling towers water systems, we chose cooling towers in 22 factories in Shijiazhuang City, Hebei province, China as study points for 17 months. The species and **s**erogroups of *Legionella* isolates were determined. Genetic characteristics were analyzed using pulsed-field gel electrophoresis (PFGE) and sequence-based typing (SBT) methods. Furthermore, two virulence genes, *lvh* and *rtxA*, which could be used as indicators of infection potential for *L. pneumophila* isolates, were examined [17].

## 2. Materials and Methods

### 2.1. Sample Collection

We selected cooling towers in 22 factories in Shijiazhuang City, China, as study points. Sodium hypochlorite, chlorine dioxide, glutaraldehyde and isothiazolin-one were used as disinfection/biocidal treatment of cooling tower water in these factories every week in the summer and every month in other seasons. The samples were taken at the same time before the disinfection/biocidal treatment. Among them cooling towers in one factory (factory A) were selected for sampling in March, April and July in 2011 and every month from October 2011 to September 2012. Cooling towers in other 21 factories (factory B–factory V) were irregular selected for sampling between March 2011 and September 2012. All 255 cooling tower water samples were collected during the study period, and the procedure for collection and pretreatments of the environmental water samples was based on the protocol according to ISO 11731 [18]. Five hundred milliliters of each water sample was collected from a reservoir or condensation pan and placed in a sterile, screw-capped container. Chlorine in the water samples was inactivated by the addition of sodium thiosulfate.

### 2.2. Legionella Detection

To detect *Legionella*, 500 milliliters of the water samples were filtrated through a membrane with 0.22-μm holes and the membranes were resuspended in 5 mL of distilled water. The resuspension were serially diluted 10-fold with sterile water. Diluted and undiluted samples (100 μL each) were plated onto buffered charcoal yeast extract (BCYE) supplemented with glycine (3 g/L), vancomycin (1 mg/L), polymyxin B (80,000 UI/L) and cycloheximide (80 mg/L) (GVPC) agar (Oxoid, Hampshire, United Kingdom), and the plates were incubated at 37 °C for 10 days. Colonies were identified as *Legionella* using the Gram staining, L-cysteine requirement test and slide agglutination using polyclonal antisera (Denka Seiken, Tokyo, Japan).

### 2.3. PFGE

One-day, standardized PFGE protocol was used for *L. pneumophila* subtyping [19]. Cell suspensions were prepared with optical density of 3.8–4.0 using a Densimat photometer (BioMérieux, Lyon, France). A 400-μL aliquot of the adjusted cell suspension was transferred to a 1.5-mL centrifuge tube and 20 μL of proteinase K (20 mg/mL stock; Invitrogen, Carlsbad, CA, USA) was added and mixed gently with the pipette tip. Plugs were made by adding an equal volume (400 μL) of molten 1.0% SeaKem Gold (SKG, Cambrex, Rockland, ME, USA) agarose to each cell suspension and the mixture was immediately dispensed into the wells of a reusable plug mold (Bio-Rad Laboratories, Hercules, CA, USA). The plugs were transferred into 50-mL polypropylene tubes containing 5 mL of cell lysis buffer (50 mM Tris, 50 Mm Ethylene Diamine Tetraacetie Acid (EDTA) (pH 8.0), 1% sarcosine, and 0.5 mg of proteinase K/mL). Lysis of cells in the plugs was performed for 2 h at 54 °C in a water bath with constant agitation (160 rpm). The plugs were washed two times with 15 mL of sterile ultrapure water and four times with 15 mL of TE buffer (10 mM Tris: 1 mM EDTA (pH 8.0)) at 50 °C. *Legionella* slices were digested using 30 U per slice of *Asc*I (New England Biolabs, Ipswich, MA, USA) for 4 h at 37 °C. Electrophoresis was run with a switch time of 6.8 s to 54.2 s for 19 h in a CHEF-DRIII system (Bio-Rad Laboratories, Hercules, CA, USA). Images were captured using a Gel Doc 2000 system (Bio-Rad) and converted to TIFF files and analyzed using the BioNumerics version 5.1 software (Applied Maths, Kortrijk, Belgium). Similarity analysis of the PFGE patterns was performed by calculating the Dice coefficients (SD) [20] and clustering was created using the unweighted-pair group method with average linkages (UPGMA).

### 2.4. SBT and Allelic Diversity Analyses

Genotyping was conducted via the standard, sequence-based typing (SBT) method of the European Working Group for *Legionella* Infections (EWGLI) using 7 genes (*flaA*, *pilE*, *asd*, *mip*, *mompS*, *proA* and *neuA*) [21,22]. The SBT database that was available on the EWGLI website [23] was used for nucleotide analysis, and the sequences were compared with those in the SBT database, which were also available on the website [24]. 

The clonal complexes were analyzed using the eBURST V3 software [25,26], and clusters of related sequence types (STs) that descended from a common ancestor were defined as clonal groups (CGs). Single genotypes that corresponded to no CG were defined as singletons, and the BioNumerics software was used to create a minimum spanning tree. In the minimum spanning tree, the founder ST was defined as the ST with the greatest number of single-locus variants. Types are represented by circles, and the size of a circle indicates the number of strains of this particular type. Heavy solid lines connect two types that differ within a single locus; light solid lines connect double-locus variants; heavy dotted lines connect triple-locus variants; light dotted lines connect quadruple-locus variants; and gray circles represent STs that are not part of any clonal complex.

### 2.5. Detection of Virulence Genes

*lvh* and *rtxA* genes were examined by PCR. The primers and PCR reaction conditions used to detect *lvh* and *rtxA* regions were same as in those described by previous studies [27].

## 3. Results

### 3.1. The Degree of Pollution of Legionella in Cooling Tower Water Samples

All 255 cooling tower water samples were tested, and *Legionella* was cultured from 91 of those samples. Different serogroups were detected from 30 samples. Viable counts for the positive samples (by using the Gram staining, L-cysteine requirement test and slide agglutination using polyclonal antisera) ranged from 100 CFU/liter to 88,000 CFU/liter with an average of 9100 CFU/liter. Overall, the data presented a positive rate of *Legionella* of 35.7% (91/255) in the cooling tower water samples of this study. Of the positive samples, 71.8% were isolated over 1000 CFU/liter. Twelve of the 22 studied factories were positive for *Legionella* in at least one sample during the study period. 

**Figure 1:**
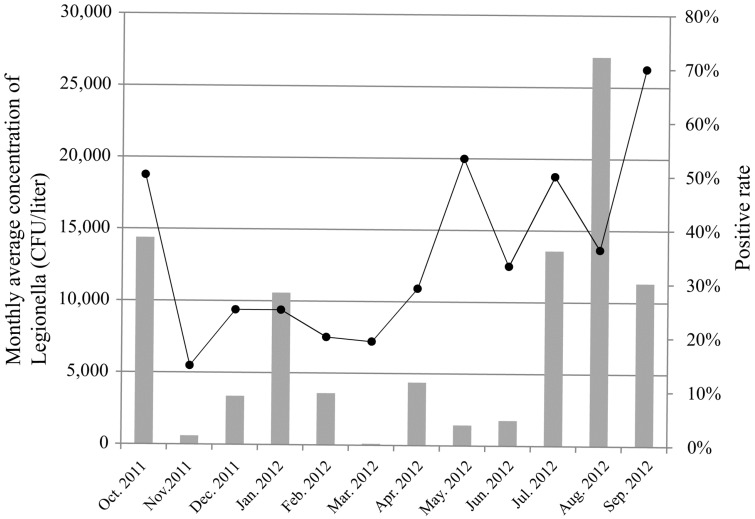
The positive rate and monthly average concentration of *Legionella* of factory A between October 2011 and September 2012. The curve represents the positive rate, and the histogram represents the monthly average concentration.

For factory A, continuous sampling of one-year period every month from October 2011 to September 2012 was carried out (Figure 1). The positive rates of *Legionella* were higher during the summer and autumn (from May to October) than during the winter (from December to April). The mean number of *Legionellae* detected varied with seasons. The lowest concentrations of *Legionella* were observed in March (100 CFU/liter); the concentrations in April, May, June were 4383 CFU/liter, 1425 CFU/liter, 1766 CFU/liter respectively; and then raised gradually in July and August, up to 13,580 CFU/liter and 27,100 CFU/liter respectively; the concentrations in October 2011 was 14,386 CFU/liter.

### 3.2. Distribution of Species and Serogroups of Legionella Isolates

We selected one to three isolates from each water sample to determine their species and serogroups. In total, 121 isolates of *Legionella* from 91 samples were obtained. All the 121 isolates were *L. pneumophila.* There were 30 samples containing two different *L. pneumophila* serogroups in each. Among the *L. pneumophila* 121 isolates, serogroup 1 was the most frequently isolated serogroup and made up 56.2% (68/121) of the strains that were isolated. The other 53 isolates belonged to serogroups 6 (25 isolates, 20.7%), 5 (12, 9.9%), 8 (8, 6.6%), 3 (6, 5.0%) and 9 (2, 1.6%). No non-*L. pneumophila* species isolate was identified.

**Figure 2:**
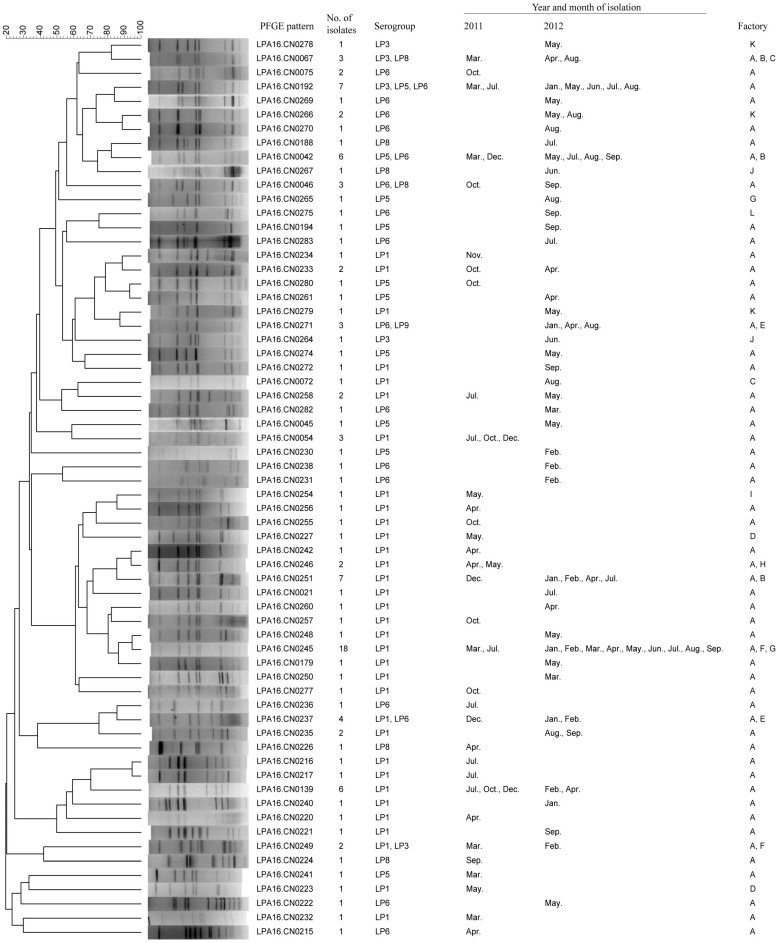
Clustering results of patterns obtained by pulsed-field gel electrophoresis (PFGE) analysis of 121 *Legionella pneumophila* strains. These strains were isolated from industrial cooling towers in Shijiazhang, China during the 19-month study period.

### 3.3. PFGE and SBT Analysis of Legionella Isolates

All 121 isolates were analyzed by PFGE, and divided into 64 different patterns (Figure 2). The pattern LPA16.CN0245 was the most frequently occurring pattern and contained 18 isolates that were isolated from three factories over 19 months. Seventeen patterns contained more than one isolate, and among these, 7, 16 and 8 patterns contained isolates from different serogroups, months and factories, respectively. Forty-four patterns contained only one isolate. These results indicated a high genetic polymorphism among these tested isolates. 

For SBT, full 7-allele profile was obtained of 117 isolates. One hundred and seventeen isolates were differentiated by SBT into 49 different sequence types (STs). Fifteen profiles (ST1, ST59, ST60, ST154, ST296, ST345, ST583, ST595, ST949, ST986, ST1177, ST1410, ST1434, ST1471 and ST1497) could be found in the EWGLI SBT database, but the profile of other 34 STs could not be found in the database. ST1 was the most frequently occurring ST and contained 39 isolates.

**Figure 3:**
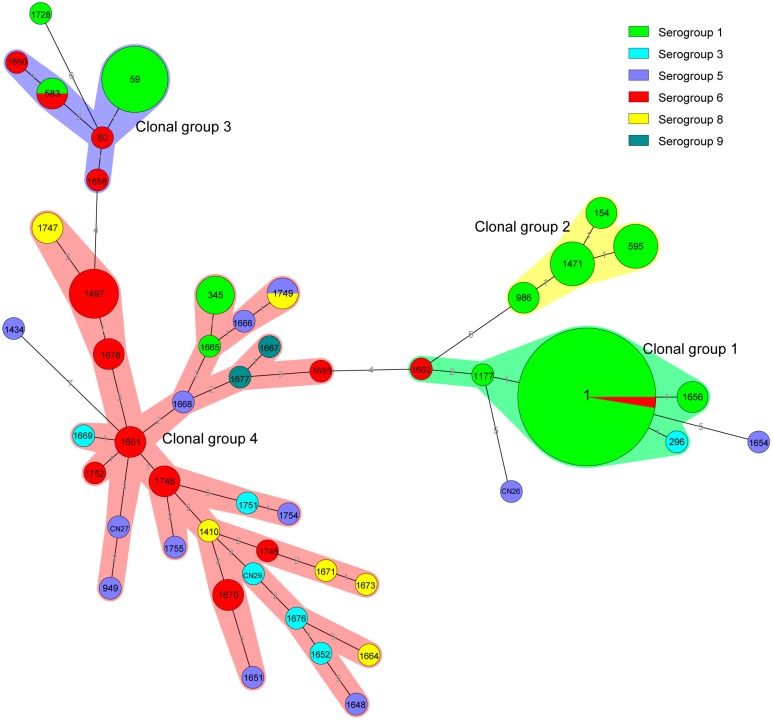
Minimum spanning tree analysis of 117 *Legionella pneumophila* isolates from industrial cooling towers in Shijiazhang, China. In the minimum spanning tree, the sequence types (STs) are displayed as circles. The size of each circle indicates the number of isolates within this particular type, and the STs are shown in the circles. The colors of the halo surrounding the STs denote types that belong to the same clonal group.

According to the results of our eBURST analysis, 49 STs belonged to 4 CGs and 4 singleton STs (Figure 3). Of the 4 CGs, CG1, which was the prevalent clonal group, included 44 isolates that belonged to ST1, ST296, ST1177, ST-1656 and ST-1662; and the putative ancestor of CG1 was predicted to be ST1. CG4 was the second prevalent clonal group, included 43 isolates that belonged to 31 STs. The reminders were CG3 (ST59, ST60, ST583, ST-1658 and ST1660), which contained 14 isolates; CG2 (ST154, ST595, ST986 and ST1471), which contained 12 isolates. Of the 4 singleton STs, each were detected in one isolate.

The serogroup distribution of CGs was different. CG1 and CG2 were mainly formed by serogroup 1; 100% and 93.2% (41/44) of CG1 and CG2 isolates were serogroup 1, respectively. CG3 was formed by serogroup 1 (10 isolates) and serogroup 6 (four isolates). The serogroup distribution of CG4 was diverse; CG4 contained four serogroup 1, five serogroup 3, nine serogroup 5, 16 serogroup 6, seven serogroup 8 and two serogroup 9 isolates.

### 3.4. Distribution of Virulence Genes of Legionella Isolates

Two virulence genes, *lvh* and *rtxA*, were examined. 90.9% (110/121) of tested isolates carried both *lvh* and *rtxA.* 92.6% (112/121) and 98.3% (119/121) isolates carried *lvh* and *rtxA* respectively. Of 121 isolates, nine showed *lvh* negative and all were non-serogroup 1; they were serogroups 3 (one isoltaes), 5 (four) and 6 (four). All the nine *lvh* negative isolates were different STs; seven of them belonged to CG4 and other two were singleton. Of 121 isolates, two showed *rtxA* negative and both were serogroup 1. The STs of two *rtxA* negative isolates were ST1 (CG1) and ST583 (CG3) respectively.

## 4. Discussion

In this study, *Legionella* were detected in 35.7% of collected industrial cooling towers water samples from 22 factories during the 19-month study period, showing a high degree of pollution of *Legionella* in industrial cooling towers. The concentration of *Legionella* was high in this study, with 71.8% of the samples giving a *Legionella* concentration that exceeded 1000 CFU/liter, which was higher than that was found in cooling tower water samples in Shanghai, China [28] and in spring water samples [29,30]. The above findings revealed a high prevalence and high concentration of *Legionella* in surveyed cooling towers. *Legionella* widely and long-standingly inhabits industrial cooling towers water in Shijiazhuang.

The *Legionella* pollution and concentration were different between seasons in this study. The positive rates were higher during the summer and autumn than that during the winter. The highest concentration was observed in the autumn. Such a seasonal variation may be due to the fact that *Legionella* multiply faster in the warmer waters of summer and autumn. In Shijiazhuang, the air temperatures were higher in the summer and autumn than other seasons. The seasonal differences of *Legionella* pollution and concentration in cooling tower water observed in this study were opposite to that in spring water samples as observed in our previous study [30]. Air temperature and utilization rate may explain the differences in the presence of *Legionella* between seasons. In the study area, air temperature in summer (30 °C–36 °C) and autumn (25 °C–30 °C) is higher than that of other seasons (−5 °C–24 °C) and could impact the water temperature of cooling towers. The cooling tower water temperatures in summer and autumn are up to 32 °C, and in other seasons is no more than 25 °C. We speculate that air temperature was one factor causing higher *Legionella* pollution and concentration of cooling towers in summer and autumn. Another factor, utilization rate, may also explain the differences in the presence of *Legionella* between seasons. Higher utilization rate of cooling towers was observed in summer and autumn that in winter; in contrast, the numbers of passengers visiting hot spring were higher in spring and winter than in summer and autumn. Based on our results of this study and a previous one [30], we suggest that more disinfection measures are needed for spring water systems in spring and winter, and more disinfection measures are needed for cooling tower water systems in summer and autumn.

All the strains isolated in this study were *L. pneumophila*. This was different to that of cooling tower water in other countries and other types of water samples in China. *L. pneumophila* accounted for 76.2% of the strains that were isolated from cooling tower water samples in South Korea*,* and this organism was predominantly found in other types of water samples, such as water from buildings, public baths, hospitals and hotels [31]. In the previous studies of cooling tower and spring water samples, other species, *Legionella micdadei* and *Legionella bozemanii*, were also isolated. In this study, serogroup 1 was the most frequently *L. pneumophila* isolate, which was in agreement with that of previous studies of cooling towers in China and other countries [28,31]; however, *L. pneumophila* serogroup 1 was not predominant in springs in China as described in previous study [30]. This observation suggested that the serogroups may have adaptability of specific kinds of water environment. The adaptability mechanism of serogroups to specific environment should be studied. 

PFGE results showed that PFGE patterns of the tested strains were highly diverse. More than one-third of isolates had a unique PFGE pattern. There were some patterns containing isolates from different serogroups, months and factories. However, the most dominant pattern persisted in the investigated cooling towers throughout the 19-month study period, suggesting that although high genetic polymorphism was displayed, a dominant pattern also existed. However, the PFGE patterns of the tested strains in this study were different to these clinical isolates of persons with diagnosed *L. pneumophila* pneumonia from China (data not shown).

SBT results showed that the population of STs was highly diverse, suggesting high genetic polymorphism. One hundred and seventeen isolates were divided into 49 STs, of which 34 STs had new allelic profiles. After querying the SBT database, we found five STs (ST1, ST59, ST60, ST345, ST595) that had previously caused Legionnaires’ diseases worldwide. In our previous study, we reported a Legionnaires’ disease that was caused by a *L. pneumophila* strain of ST59, which was the second predominant ST in this study [32]. ST1, which is the most prevalent ST worldwide [33,34,35,36,37,38,39], was the most predominant ST in this study. Similar results have been reported in other countries. In Japan, the majority (29%) of environmental isolates, especially from cooling tower waters (74%), were ST1 [33]. In our previous study focusing on the ST distribution of *L. pneumophila* serogroup 1 strains isolated from different type of water samples in China, results showed that ST1 was the predominant type, accounting for nearly half of the analyzed strains [40]. *L. pneumophila* serogroup 1 and ST1 are the predominant serogroup and ST caused clinical Legionnaires’ disease cases, so the *Legionella* isolates in cooling towers surveyed in this study have the potential to caused *Legionella* infections and Legionnaires’ disease outbreaks. 

*lvh* and *rtxA* are two virulence genes of *L. pneumophila* [41,42]. So, a PCR detection test strategy with *lvh* and *rtxA* as pathogenesis markers would be useful for determining the infection potential of an isolate (17). In this study, 90.9% of *L. pneumophila* isolates, regardless of the serogroups, carried both *lvh* and *rtxA*; which further suggests the infection potential of *L. pneumophila* isolates in cooling tower waters.

## 5. Conclusions

Our results demonstrated high prevalence and genetic polymorphism of *Legionella* in industrial cooling towers. Furthermore, the serogroup, SBT and virulence genes detection results suggested that the *Legionella* isolates of industrial cooling tower environments may be pathogenic. Improved control and prevention strategies of *Legionella*, containing routine monitoring of industrial cooling tower water samples, using more detection technologies such as PCR, qPCR and EMA-qPCR, for detection of viable but nonculturable (VBNC) cells and to overcome underestimation by culture methods, as well as monitoring of *Legionella*-containing aerosols, are urgently needed.
